# Diagnostic and Predictive Features of Pancreatic Cysts Using Endoscopic Ultrasound: A Retrospective Analysis from a Single Centre

**DOI:** 10.5152/tjg.2025.24486

**Published:** 2025-06-23

**Authors:** Ayşegül Dumludağ, Mehmet Cindoruk

**Affiliations:** 1 Department of Internal Medicine, Division of Medical Oncology, Erzurum City Hospital, Erzurum, Türkiye; 2 Department of Internal Medicine, Division of Gastroenterology,Gazi University Faculty of Medicine, Ankara, Türkiye

**Keywords:** Pancreatic cyst, endosonography, diagnostic, predictive, malignancy

## Abstract

**Background/Aims:**

Endoscopic ultrasound (EUS) has become an increasingly important tool in modern medicine, particularly in the diagnosis and treatment of pancreatic pathologies. The aim of this study is to elucidate the diagnostic and predictive roles of EUS in pancreatic cysts.

**Materials and Methods:**

Patients who underwent EUS for various clinical indications were retrospectively analyzed. Among them, the detailed characteristics of pancreatic cysts identified during the procedures were documented from the patients’ medical records.

**Results:**

A total of 1146 patients were included in the study, with a mean age of 56.4 years; 51.7% were female and 48.3% were male. Endoscopic ultrasound was primarily used to evaluate pancreatic lesions, focusing on cysts. Of a total of 200 pancreatic cysts evaluated pathologically, 74% (148) were benign, 24.5% (49) were malignant, and 1.5% (3) were borderline. No significant correlation was found between cyst size and malignancy (*P* = 1.00). However, malignancy rates significantly varied according to ultrasonographic characteristics, including simple cysts, mixed-type cysts (with solid components), pseudocysts, and infected cysts. The malignancy rate was significantly higher in mixed-type cysts (with solid components) (*P* = .0001). The presence of cyst-associated lymphadenopathy was also statistically significant for suspicion of malignancy (*P* = .005).

**Conclusion:**

This study highlights the importance of early diagnosis or surgical intervention for pancreatic cysts with mixed-type characteristics and associated lymph nodes. This underscores the need to prioritize triage for these patients.

**Main Points**

- Endoscopic ultrasound is a critical diagnostic tool for pancreatic cysts, offering high sensitivity and specificity in distinguishing benign, malignant, and borderline lesions.
- Mixed-type cysts exhibit significantly higher malignancy rates compared to other cyst types (*P* = .0001), highlighting the necessity for close monitoring and early intervention.
- Lymphadenopathy associated with pancreatic cysts is a significant predictor of malignancy, with its presence strongly correlating with malignant potential (*P* = .005).
- Cyst size does not significantly correlate with malignancy risk, suggesting that size alone should not be the primary criterion for assessing malignant potential.
- Endoscopic ultrasound is increasingly recommended as a primary diagnostic tool for pancreatic lesions, often used in conjunction with other imaging modalities to improve the diagnostic accuracy and guide treatment strategies.

## Introduction

Pancreatic cysts are frequently encountered lesions in clinical practice. Since they are mostly asymptomatic, they are often incidentally detected during imaging studies performed for other reasons. Among these imaging modalities, endoscopic ultrasonography (EUS), magnetic resonance imaging (MRI), and computed tomography are commonly used. The prevalence of pancreatic cysts increases with age. These cysts can be neoplastic or non-neoplastic, and this distinction is crucial in determining the follow-up and treatment strategies for patients.

Although imaging methods provide diagnostic clues, the overlapping radiological features of these lesions make the precise characterization of cysts challenging. This highlights the critical role of accurately identifying specific cyst types in the clinical decision-making process. In this context, EUS stands out as an important diagnostic tool for the evaluation of pancreatic cysts. EUS not only allows for the assessment of macroscopic and microscopic features of cysts but also enables chemical and molecular analysis of cyst fluid. Additionally, it offers significant advantages for performing therapeutic interventions. In cases where findings suggest malignancy, rapid triage with EUS and the possibility of early intervention play a pivotal role in improving patient prognosis.

However, despite the currently known characteristics of pancreatic cysts and available diagnostic procedures, there is no definitive method to conclusively determine whether these cysts are benign or malignant. This study aims to identify predictive features of malignancy in pancreatic cysts using EUS imaging and cyst fluid analysis and to contribute these findings to the literature. By doing so, it is hoped that more effective strategies for the diagnosis and management of pancreatic cysts can be developed.^1^^-^^6^

## Materials and Methods

This retrospective study was conducted on patients who underwent EUS between September 2011 and September 2016.

Patients were included based on the presence of pancreatic cysts identified during EUS procedures. Inclusion criteria comprised the availability of complete medical records, confirmed pancreatic cyst diagnosis via EUS, and an age of 18 years or older. Patients with incomplete data or those who underwent EUS for non-pancreatic indications were excluded from the study.

Data were collected from patient records, focusing on demographic information such as age and gender, as well as clinical indications for EUS. Detailed cyst characteristics, including size, number (single or multiple), septation, ductal connection, and the presence of mural nodules, were documented. Ultrasonographic features such as simple cysts, mixed-type cysts, pseudocysts, and infected cysts were evaluated, along with the presence of associated lymphadenopathy (LAP). Pathological outcomes were categorized as benign, malignant, or borderline based on histopathological or cytological examination. Additionally, cyst fluid analysis parameters, including carcinoembryonic antigen (CEA) levels, amylase levels, and string sign positivity, were recorded.

Statistical analysis was performed using SPSS software version 24.0 (IBM SPSS Corp.; Armonk, NY, USA). Descriptive statistics were presented as mean ± SD for continuous variables and percentages for categorical variables. The chi-square test was employed for cross-comparisons of categorical variables, with a *P*-value < .05 considered statistically significant.

Ethical approval for this study was granted by the Local Ethics Committee of Gazi University on February 11, 2017 (Document No: E.37466). As the study was retrospective and utilized anonymized patient data, informed consent was not required.

## Results

In this retrospective study, it was determined that 1462 patients underwent EUS within the specified time period. However, due to data loss and difficulties in accessing patient information, data were collected from 1146 patients for analysis.

The average age of these 1146 patients was 56.4, with ages ranging from 18 to 91 years. The cohort consisted of 51.7% (592) female and 48.3% (554) male patients. Endoscopic ultrasound procedures were primarily performed on individuals under the age of 50, accounting for 31.7% (363) of cases. In this subgroup, 55.4% (201) were female, and 44.6% (162) were male.

Analysis of the indications for EUS procedures revealed that the most frequent reasons were pancreatic lesions, including cysts and masses, as well as lesions involving the stomach and duodenum (Table 1). Among patients undergoing EUS for suspected pancreatic cysts, 156 were confirmed to have pancreatic cysts. Additionally, pancreatic cysts were incidentally detected in 44 patients undergoing EUS for other indications. In patients specifically evaluated for pancreatic cysts, 16% (32) had pancreatic masses, 78% (156) had pancreatic cysts, 3.5% (7) had autoimmune or chronic pancreatitis, 2% (4) had biliary tract diseases, and 0.5% (1) had extrinsic compression.

The average size of pancreatic cysts was 40.8 ± 14 mm, with a median cyst size of 26 mm, and cyst sizes ranged from 4 to 371 mm. Among the 200 patients with pancreatic cysts, 79.5% (159) had a single cyst, while 20.5% (41) had multiple cysts. Of these cysts, 73.2% (145) were non-septated, and 84.5% (169) were larger than 1 cm. Specifically, 73.2% (145) of the cysts were septated, while 26.8% (53) were non-septated. An analysis of cyst types revealed that 33% (66) were simple cysts, 13% (26) were infected, 14.5% (29) were pseudocysts, and 39.5% (79) had mixed characteristics (including solid components).

Regarding ductal dilation, 83% (166) of patients did not exhibit ductal dilation, while 17% (34) did. In terms of cyst-associated LAP, 83% (166) of patients did not have LAP, 11% (22) had LAP, and in 6% (12) of patients, LAP status was unknown. The images of patients with LAP were confirmed through tomography, MRI, and MRCP (Magnetic resonance cholangiopancreatography), with an average size of 17 ± 5.3 mm. Of these, 15 exhibited benign characteristics, while 7 had malignant features. For patients with malignant LAP, fine-needle aspiration (FNA) was performed in 5 patients, while 2 were inaccessible due to anatomical reasons. The distribution of LAP by location was as follows: 9 cases in the celiac region, 3 in the pancreatosplenic, 3 in the pancreaticoduodenal, 2 in the hepatic, 2 in the right gastric, 1 in the left gastric, 1 in the phrenic, and 1 in the superior mesenteric nodes.

Regarding cyst pathology, 48 were malignant, 148 were benign, and 4 were borderline. Of the malignant cysts, 39.5% (19) had mural nodules. When examining the relationship between cysts and the pancreatic duct, 80% (160) of cysts were not connected to the duct, while 17.5% (35) were connected. Among benign cysts, pseudocysts, serous cystadenomas, and mucinous cystadenomas were the most common types (Table 2). Among malignant cysts, the most common pathology was pancreatic adenocarcinoma. The distribution of EUS images for cysts with malignant pathology was as follows: serous cystadenoma (2 cases, 4%), mucinous cystadenoma (10 cases, 20%), intraductal papillary mucinous neoplasm IPMN (intraductal papillary mucinous tumor) (7 cases, 14%), rare tumors (neuroendocrine tumor, 1 case, 2%), and pancreatic adenocarcinoma (26 cases, 52%).

Regarding the need for surgical intervention, 67% (134) of patients did not require surgery, 20% (40) required surgery, and in 13% (26) of patients, there was no information regarding surgical management. Among the 40 patients who underwent surgery, 12 had LAP excision, 10 of which were malignant and 2 were benign. Regarding FNA for pancreatic cysts, 108 patients underwent FNA, while 92 did not. The reasons for not performing FNA are given in detail below (Table 3).

Records of string signs for the 108 patients who underwent FNA were examined. A string formation lasting at least 1 second and 1 cm in length was defined as a positive sign. The string sign was found to be positive in 9 out of 17 patients (52.9%) with mucinous-looking malignancy (*P* = .0001). Regarding CEA levels in cyst fluid, the cutoff value recommended by current guidelines (>192 ng/mL) was applied. In the mucinous cystic neoplasm group, the average CEA value was 789 ± 882 ng/mL, while in non-mucinous neoplasms, it was 132 ± 246 ng/mL (*P* = .02). No significant difference in amylase levels was found between the group with a positive string sign (36 737 ± 110 505 units/liter) and the group with a negative string sign (32 303 ± 54 009 units/liter) (*P* = .5).

The correlation between cyst size and malignancy was examined, and no significant relationship was found. Additionally, the relationship between the number of cysts, their connection to the pancreatic duct, and ductal dilation was evaluated, showing no significant difference between single and multiple cysts in these aspects.

**Figure 1.**
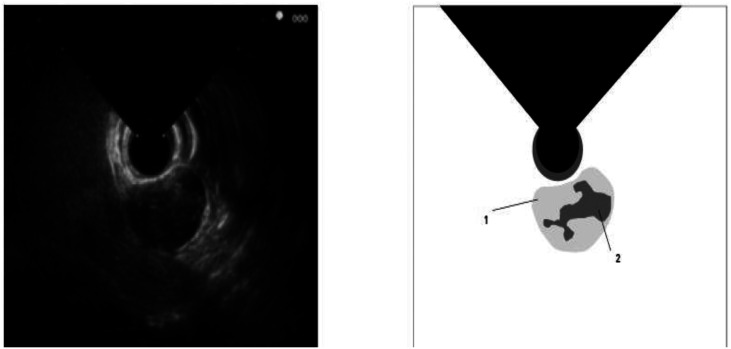
Cystic lesion in the mid-pancreatic region containing necrotic material with a hyperechoic appearance. 1: Pancreatic cyst 2: Necrotic material.

A more detailed examination of cyst characteristics based on numbers yielded noteworthy findings. Simple cysts showed malignant features in 12.5% (8) of cases, while 87.5% (56) were classified as benign. In contrast, 17.4% (6) of the 29 cases interpreted as pseudocysts in EUS images exhibited malignant features, and 82.6% (23) were benign. Among infected cysts with debris (26 cases), 3.8% (1) were malignant, and 96.2% (25) were benign (Figure 1). Mixed-type and honeycomb-appearing cysts showed a distribution of 42.9% (33) malignant and 57.1% (44) benign, with statistical significance (*P* < .05) (Table 4).

When comparing the presence or absence of lymphadenopathy with pathological outcomes, a statistically significant relationship was observed, indicating that lymphadenopathy was more frequently associated with malignant cysts (*P* = .005) (Table 5). Statistical analyses did not reveal significant differences in pathological outcomes based on gender and age. A total of 3 (0.26%) complications were observed during EUS procedures. Specifically, 1 patient experienced perforation, while 2 patients developed hypoxia, particularly related to anesthesia. Thus, it was concluded that only 1 patient experienced a complication directly related to the EUS procedure.

## Discussion

This study highlights the critical role of EUS in the diagnostic evaluation of pancreatic cysts. Among the key findings, mixed-type cysts with solid components demonstrated significantly higher malignancy rates compared to other cyst types, underscoring the necessity for close monitoring and early intervention in such cases. Additionally, the presence of cyst-associated LAP was strongly correlated with malignant potential (*P* = .005), further supporting its use as a predictive marker for malignancy. Interestingly, cyst size did not show a significant correlation with malignancy risk, suggesting that size alone should not be the primary criterion for assessing malignant potential. These findings collectively emphasize the importance of EUS in guiding clinical decision-making and optimizing patient outcomes.

One of the strengths of this study is its comprehensive analysis of a large patient cohort, which provides robust and generalizable data. The detailed characterization of cyst features, including ultrasonographic morphology, cyst fluid biomarkers (e.g., CEA levels, string sign), and pathological outcomes, offers valuable insights into the diagnostic and predictive utility of EUS. Furthermore, the integration of multiple imaging modalities and standardized diagnostic protocols strengthens the reliability of the conclusions. These aspects collectively contribute to the growing body of evidence supporting EUS as an indispensable tool in the management of pancreatic cysts.

However, this study has certain limitations. Its retrospective design may introduce selection bias, and the reliance on historical patient records could result in incomplete data for certain variables. Moreover, the absence of molecular analyses represents a missed opportunity to further elucidate the biological behavior of pancreatic cysts. Despite these limitations, these findings provide significant contributions to the field, particularly in identifying high-risk cyst features and optimizing patient triage.

In this study, the presence of mural nodules was observed in 38.7% of the cysts diagnosed with malignancy, highlighting its importance as a predictive feature.^8^ Additionally, high levels of CEA in cyst fluid analysis obtained by FNA were identified as a significant finding in malignant cysts. Although there is no consensus in the literature regarding the minimum length for considering the string sign as positive when correlated with CEA, the positivity of the string sign is evaluated as a significant parameter in mucinous pancreatic cysts, and it is suggested that this test should be performed.^9^^,^^10^

The detection of malignancy in 23.1% of pseudocysts exhibiting benign features is an important finding that should be considered in the follow-up of pseudocysts.^11^ Similarly, the development of malignancy in 3.6% of infected cysts with debris makes regular follow-up of these patients mandatory. These findings underscore the complexity of pancreatic cysts and the need for careful evaluation and monitoring.

In conclusion, this study reaffirms the critical role of EUS in the evaluation of pancreatic cysts, particularly in identifying high-risk features such as mixed-type morphology, cyst-associated LAP, and mural nodules. These findings have important implications for clinical practice, enabling more accurate risk stratification and timely intervention. Future research incorporating molecular profiling and prospective study designs is warranted to further refine diagnostic algorithms and improve patient outcomes in the management of pancreatic cysts.^7^^,^^12^^,^^13^

## Figures and Tables

**Table 1.** Distribution of Indications for the Total Patients Participating in the Study

Indication Groups	Number	%
Pancreatic mass	207	18.1
Pancreatic cyst	183	16
Chronic pancreatitis/autoimmune	68	5.9
Periampullary/anuler duct dilation	32	2.8
Esopagheal disease	127	11.1
Stomach and duodenum	331	28.9
Gastric fold	4	0.3
Mediastinal lenfadenopathy/lung mass	10	0.9
Bile duct disease	115	10
Other (CA19-9, abdominal lenfadenopathy)	23	2
Rectal disease	8	0.7
Celiac blockage	2	0.2
External compression	35	3.1

**Table 2.** Benign Pancreatic Cysts

Distribution of Pancreatic Cysts (n = 148)	Number	%
Pseudocyst	81	54.7
Serous cystadenoma	34	22.9
Mucinous cystadenoma	10	6.7
IPMN	4	2.7
Polycystic disease	4	2.7
Solid pseudopapillary neoplasm	4	2.7
Infectious	3	2.0
Postnecrotic fluid collection	3	2.0
Hematoma	1	0.6
Postinflammatory fluid collection	4	2.7

**Table 3.** The Situation of Performing Fine Needle Aspiration on Pancreatic Cysts

	Number	%
The Condition for Performing FNA (n = 200)		
No FNA	92	46
FNA present	108	54
**Reasons for the inability to perform FNA (n = 92)**		
Small size	8	8.6
Distant location	11	11.9
The diagnosis is sufficient	18	19.5
No explanation provided	28	30.4
Related to characteristic of cyst	5	5.4
Hypervascularisation	2	2.1
Being close to blood vessels	10	10.8
Technical reasons	10	10.8

FNA, fine needle aspiration.

**Table 4.** Comparison of Cyst Features with Pathology

Cyst Feature	Pathology			
	Malignant		Benign	
	Number	%	Number	%
Simple	8	12.5	56	87.5
Pseudocyst	6	23.1	23	76.9
Infected	1	3.8	25	96.2
Mixed	33	42.9	44	57.1

χ^2^ = 25.586.

*P = .0001.

*Yates correction has been applied.

**Table 5.** Relationship Between the Presence of Lymphadenopathy and Pathology

The Presence of Lymphadenopathy	Pathology			
	Malignant		Benign	
	Number	%	Number	%
**No lymphadenopathy**	15	31.2	130	87.8
Lymphadenopathy present	33	68.7	18	12.2

χ. ^2^ = 7.897.

*P = .005.

*Yates correction has been applied.

## References

[1] Wesali S Molinaro A Lindkvist B Hedenström P Sadik R. Endoscopic ultrasound is useful for the risk stratification of mucinous pancreatic cystic lesions: a long-term prospective study. Pancreatology. 2024:S1424-3903(24)00834-2. (doi: 10.1016/j.pan.2024.12.006) 39741057

[2] Lennon AM Vege SS. Pancreatic cyst surveillance. Clin Gastroenterol Hepatol. 2022;20(8):1663 1667.e1. (doi: 10.1016/j.cgh.2022.03.002) 35397230 PMC10548438

[3] Singh S Chandan S Vinayek R Endoscopic techniques for the diagnosis of pancreatic cystic lesions. World J Gastroenterol. 2025;31(1):101082. (doi: 10.3748/wjg.v31.i1.101082) PMC11684177 39777250

[4] Ohno E Kuzuya T Kawabe N Current status of endoscopic ultrasound in the diagnosis of intraductal papillary mucinous neoplasms. DEN Open. 2025;5(1):e413. (doi: 10.1002/deo2.413) PMC11260769 39040523

[5] Öztürk B Ceyhan K Bektaş M. The effectiveness of endoscopic ultrasonography and computed tomography in the differentiation of pancreatic cystic neoplasms: a single-center experience. Turk J Gastroenterol. 2024;35(12):945 953. (doi: 10.5152/tjg.2023.23492) 39641318 PMC11639599

[6] Syed KA Lafaro KJ Afghani E. Current endoscopic and surgical management of pancreatic cystic lesions: a comprehensive review. Turk J Gastroenterol. 2024;36(1):6 14. (doi: 10.5152/tjg.2024.24124) 39634190 PMC11736832

[7] Iwashita T Uemura S Mita N Utility of endoscopic ultrasound and endoscopic ultrasound-guided fine-needle aspiration for the diagnosis and management of pancreatic cystic lesions: differences between the guidelines. Dig Endosc. 2020;32(2):251 262. (doi: 10.1111/den.13579) 31709639

[8] Hong SB Lee NK Kim S Diagnostic performance of magnetic resonance image for malignant intraductal papillary mucinous neoplasms: the importance of size of enhancing mural nodule within cyst. Jpn J Radiol. 2022;40(12):1282 1289. (doi: 10.1007/s11604-022-01312-y) 35781178

[9] Sbeit W Kadah A Shahin A Khoury T. The yield of string sign in differentiating mucinous from non-mucinous pancreatic cysts: A retrospective cross-sectional study. Medicina (Kaunas). 2021;57(7):716. (doi: 10.3390/medicina57070716) PMC8305072 34356997

[10] Hakim S Coronel E González GMN An international study of interobserver variability of “string sign” of pancreatic cysts among experienced endosonographers. Endosc Ultrasound. 2021;10(1):39 50. (doi: 10.4103/eus.eus_73_20) 33473044 PMC7980687

[11] Sedhai S Mohammed F Sahtiya S Pancreatic neuroendocrine tumor (PNET) presenting as a pseudocyst: a Case report. Cureus. 2022;14(9):e29617. (doi: 10.7759/cureus.29617) PMC9603887 36320996

[12] Morgell A Reisz JA Ateeb Z Metabolic characterization of plasma and cyst fluid from cystic precursors to pancreatic cancer patients reveal metabolic signatures of bacterial infection. medRxiv [preprint]. J Proteome Res. 2020:2020.11.03.20225524. Update in: J Proteome Res. 2021;20(5):2725 2738. (doi: 10.1021/acs.jproteome.1c00018) 33720736

[13] Nikiforova MN Wald AI Spagnolo DM A combined DNA/RNA-based next-generation sequencing platform to improve the classification of pancreatic cysts and early detection of pancreatic cancer arising from pancreatic cysts. Ann Surg. 2023;278(4):e789 e797. (doi: 10.1097/SLA.0000000000005904) 37212422 PMC10481930

